# Supplementary material for: Analysis of Metabolites in Gout: A Systematic Review and Meta-Analysis
Source: Nutrients. 2023 Jul 14;15(14):3143. doi: 10.3390/nu15143143 (PMC10383779; doi:10.3390/nu15143143)
Supplement: Supplementary file 1 [file nutrients-15-03143-s001.zip › nutrients-2490525-supplementary.pdf]

# Supplemental File

Table S1 Search strategy
Table S2 The molar mass of each bile acid for transforming unit.
Table S3 (A) Formula 1 for converting median and interquartile range (IQR) into mean and standard deviation (SD). (B) Formula 2 for combining mean and SD.
Table S4 The characteristic for each bile acids
Table S5 The NOS assessment scale for every study.

Table S1 Search strategy

| | | |
|---|---|---|
| Pubmed | # 1 | "Gout"[Mesh] |
| | #2 | (Gout[Title/Abstract]) OR (Gouts[Title/Abstract]) |
| | #3 | # 1 or #2 |
| | #4 | "Metabolomics"[Mesh] |
| | #5 | ((Metabolomic[Title/Abstract]) OR (Metabonomics[Title/Abstract])) OR (Metabonomic[Title/Abstract]) |
| | #6 | ((((((((((((((((((((Metabolomic*[Title/Abstract]) OR (Metabonomic*[Title/Abstract])) OR (Metabolit*[Title/Abstract])) OR (Metabolome[Title/Abstract])) OR (h nmr[Title/Abstract])) OR (nuclear magnetic resonance spectroscopy[Title/Abstract])) OR (proton nmr[Title/Abstract])) OR (proton nuclear magnetic resonance[Title/Abstract])) OR (gas chromatogra[Title/Abstract])) OR (gcms[Title/Abstract])) OR (gas chromatograph-mass spectrometry[Title/Abstract])) OR (gctofms[Title/Abstract])) OR (gas chromatography/time-of-flight mass spectrometry[Title/Abstract])) OR (liquid chromatogra[Title/Abstract])) OR (lc ms[Title/Abstract])) OR (triple quadrupole mass spectrometry[Title/Abstract])) OR (uplc[Title/Abstract])) OR (ultra performance liquid chromatograph[Title/Abstract])) OR (ultra-performance liquid chromatograph[Title/Abstract])) OR (high performance liquid chromatograph[Title/Abstract])) OR (ultraperformance liquid chromatography quadruple time-of-flight mass spectrometer[Title/Abstract])) OR (uhplctqms[Title/Abstract])) OR (ultra-high performance liquid chromatography triple quadrupole mass |
| | #7 | #4 or#5 or#6 |
| | #8 | #3 and #7 |
| | #9 | "Animals"[Mesh] |
| | # 10 | "Humans"[Mesh] |
| | # 11 | ("Animals"[Mesh]) NOT ("Humans"[Mesh]) |
| | # 12 | #8 not #11 |
| Embase | # 1 | 'gout'/exp |
| | #2 | gout :ti,ab,kw OR gouts :ti,ab,kw |
| | #3 | # 1 OR #2 |
| | #4 | 'metabonomics'/exp |
| | #5 | metabolomic*:ti,ab,kw OR metabonomic*:ti,ab,kw OR metabolit*:ti,ab,kw OR metabolome:ti,ab,kw OR breathomics:ti,ab,kw OR degradomics:ti,ab,kw OR fluxomics:ti,ab,kw OR metabonomics:ti,ab,kw OR nutrimetabolomics:ti,ab,kw OR 'h nmr':ti,ab,kw OR 'nuclear magnetic resonance spectroscopy':ti,ab,kw OR 'proton nmr':ti,ab,kw OR 'proton nuclear magnetic resonance':ti,ab,kw OR 'gas chromatogra':ti,ab,kw OR 'gc ms':ti,ab,kw OR 'gas chromatograph-mass spectrometry':ti,ab,kw OR 'gc tof ms':ti,ab,kw OR 'gas chromatography/time-of-flight mass spectrometry':ti,ab,kw OR 'liquid chromatogra':ti,ab,kw OR 'lc ms':ti,ab,kw OR 'triple quadrupole mass spectrometry':ti,ab,kw OR uplc:ti,ab,kw OR 'ultra performance liquid chromatograph':ti,ab,kw OR 'ultra-performance liquid chromatograph':ti,ab,kw OR 'high performance liquid chromatograph':ti,ab,kw OR 'ultraperformance liquid chromatography quadruple time-of-flight mass spectrometer':ti,ab,kw OR 'uhplc tq ms':ti,ab,kw OR 'ultra-high performance liquid chromatography triple quadrupole mass spectrometry':ti,ab,kw |

| | | |
|---|---|---|
| | #6 | #4 OR #5 |
| | #7 | #3 OR #4 |
| | #8 | #7 AND [humans]/lim |
| Web of science | # 1 | TS=(“gout” OR “gouts”) |
| | #2 | TS=(“Metabolomic*” OR “Metabonomic*” OR “Metabolit*” OR Metabolome OR “h nmr” OR “nuclear magnetic resonance spectroscopy” OR “proton nmr” OR “proton nuclear magnetic resonance” OR “gas chromatogram” OR “gc ms” OR “gas chromatograph-mass spectrometry” OR “gc tof ms” OR “gas chromatography/time-of-flight mass spectrometry” OR “liquid chromatogram” OR “lc ms” OR “triple quadrupole mass spectrometry” OR uplc OR “ultra performance liquid chromatograph” OR “ultra-performance liquid chromatograph” OR “high performance liquid chromatograph” OR “ultraperformance liquid chromatography quadruple time-of-flight mass spectrometer” OR “uhplc tq ms” OR “ultra-high performance liquid chromatography triple quadrupole mass spectrometry”) |
| | #3 | # 1 AND #2 |
| | #4 | TS=(Animals) NOT TS= (Humans) |
| | #5 | (#3) NOT #4 |
| The Cochrane Library | # 1 | MeSH descriptor: [Gout] explode all trees |
| | #2 | (Gout or Gouts):ti,ab,kw |
| | #3 | # 1 or #2 |
| | #4 | MeSH descriptor: [Metabolomics] in all MeSH products |
| | #5 | (Metabolomics or Metabonomic or Metabonomics or Metabolomic):ti,ab,kw |
| | #6 | MeSH descriptor: [Metabolomics] in all MeSH products |
| | #7 | (Metabolome or Metabolomes or Profile, Metabolic or Metabolic Profile or Metabolic Profiles or Profiles, Metabolic):ti,ab,kw |
| | #8 | MeSH descriptor: [Gas Chromatography-Mass Spectrometry] explode all trees |
| | #9 | (Gas Chromatography-Mass Spectrometry or Gas Liquid Chromatography Mass Spectrometry or Gas-Liquid Chromatography-Mass Spectrometry or Chromatography-Mass Spectrometry, Gas-Liquid or Chromatography, Gas Liquid Mass Spectrometry or Spectrometry, Gas-Liquid Chromatography-Mass or Chromatography, Gas-Liquid-Mass Spectrometry or Spectrometry-Gas Chromatography, Mass or Mass Spectrometry Gas Chromatography or Gas Chromatography Mass Spectrometry or Chromatography, Mass Spectrometry-Gas or Spectrometry, Gas Chromatography-Mass or Chromatography, Gas Mass Spectrometry or Spectrometry, Mass-Gas Chromatography or Spectrometries, Mass-Gas Chromatography or Spectrum Analysis, Mass Gas Chromatography or Chromatography-Mass Spectrometry, Gas or Chromatography, Gas-Mass Spectrometry or Spectrum Analysis, Mass-Gas Chromatography or GCMS or Spectrometry, Mass Gas Chromatography or Mass Spectrometry-Gas Chromatography):ti,ab,kw |
| | # 10 | MeSH descriptor: [Chromatography, High Pressure Liquid] explode all trees |
| | # 11 | (High-Performance Liquid Chromatographies or Chromatography, High Performance Liquid or Chromatography, High Speed Liquid or Liquid Chromatography, High-Performance or HPLC or Chromatography, Liquid, High Pressure or High Performance Liquid Chromatography or High-Performance Liquid Chromatography or Chromatography, High-Performance Liquid):ti,ab,kw |
| 知网 | # 1 | (SU %= '痛风' OR TKA= '痛风' OR SU%= '高尿酸血症' OR TKA= '高尿酸血症' OR SU %='HUA' OR TKA='HUA') AND (SU % = '代谢组学' OR TKA= '代谢组' OR TKA = 'NMR' OR TKA = '核磁共振' OR TKA= 'proton NMR' OR TKA = '质子 NMR' OR TKA = 'GC-MS' OR TKA = '气相色谱-质谱联用' OR TKA = 'GC-TOF-MS' OR TKA = '气相色谱-飞行时间质谱' OR TKA = 'LC-MS' OR TKA = '液相色谱-质谱联用' |

| | | |
|---|---|---|
| | | OR TKA= 'TQ MS' OR TKA = '三重四极杆液质联用' OR TKA= 'UPLC' OR TKA = '超高 效液相色谱' OR TKA = 'HPLC' OR TKA = '高效液相色谱' OR TKA = 'UHPLC-MS/MS' OR TKA = '超高效液相色谱- 串联质谱' OR TKA = 'UPLC-QTOF-MS' OR TKA = '超高效液相色谱- 四极 杆-飞行时间串联质谱' OR TKA = 'UHPLC-TQ-MS' OR TKA = ' 超高效液相色谱三重四极杆质谱') |
| 万方 | #1 | (主题:("痛风") or 题名或关键词:("痛风") or 主题:("高尿酸血症") or 题名或关键词: ("高尿酸血症") or 主题:("HUA") or 题名 或关键词 :("HUA")) and ( 主题 :(" 代谢组学") or 题名或关键 词 :(" 代谢组 ") or 题名或关键词 :("H NMR") or 题名或关键 词:("核磁共振") or 题名或关键词:("proton NMR") or 题名或关 键词:("质子 NMR") or 题名或关键词:("GC-MS") or 题名或关键 词:("气相-联用") or 题名或关键词:("GC-TOF-MS")or 题名或关 键词:("气相-飞行时间") or 题名或关键词:("LC-MS") or 题名或关键词:("液相-联用") or 题名或关键词:("TQ MS") or 题名或关 键词:("三重四极杆液质联用") or 题名或关键词:("UPLC") or 题 名或关键词:("HPLC") or 题名或关键词:("高效液相")or 题名或 关键词 :("UHPLC-MS/MS") or 题名或关键词:("高效- 串联") or 题名或关键词:("UPLC-QTOF-MS") or 题名或关键词:("高效- 四 极- 飞行") or 题名或关键词:("UHPLC-TQ-MS") or 题名或关键 词:("高效三重四极杆") ) |
| VIP | #1 | ((M="痛风" OR "高尿酸血症" OR "HUA") OR (R="痛风" OR "高尿酸血症" OR "HUA")) AND ((M=" 代谢组学" OR "代谢组" OR "NMR" OR "核磁共振" OR "proton NMR" OR "质子 NMR OR GC-MS" OR "气相色谱- 质谱联用" OR "GC-TOF-MS" OR "气相色谱- 飞行时间质谱" OR "LC-MS" OR "液相色谱-质谱联 用" OR "TQ MS" OR "三重四极杆液质联用" OR "UPLC" OR "超高效液相色谱" OR "HPLC" OR "高效液相色谱" OR "UHPLC-MS/MS" OR "超高效液相色谱-串联质谱" OR "UPLC-QTOF-MS" OR "超高效液相色谱- 四极杆-飞行时间串联 质谱" OR "UHPLC-TQ-MS" OR "超高效液相色谱三重四极杆质 谱") OR (R="代谢组学" OR "代谢组" OR "NMR" OR "核磁共 振" OR "proton NMR" OR "质子 NMR OR GC-MS" OR "气相色 谱-质谱联用" OR "GC-TOF-MS" OR "气相色谱- 飞行时间质谱" OR "LC-MS" OR "液相色谱-质谱联用" OR "TQ MS" OR "三重 四极杆液质联用" OR "UPLC" OR "超高效液相色谱" OR "HPLC" OR "高效液相色谱" OR "UHPLC-MS/MS" OR "超高效 液相色谱- 串联质谱" OR "UPLC-QTOF-MS" OR "超高效液相色 谱- 四极杆- 飞行时间串联质谱" OR "UHPLC-TQ-MS" OR "超高 效液相色谱三重四极杆质谱")) |

Table S2 The molar mass of each small-molecule metabolite was used for the transformed units

| | PubChem CID | molecular formula | Molecular Weight (g/mol) |
|---|---|---|---|
| Inosine | 135398641 | C10H12N4O5 | 268.23 |
| Adenosine | 60961 | C10H13N5O4 | 267.24 |

Table S3 Formula 1 for converting median and interquartile range (IQR) into mean and standard deviation (SD)(A).

| Item | Formulas |
|---|---|
| Median | Median = Mean |
| IQR | IQR = 1.35 SD |

Table S3 Formula for combining mean and SD(B).

| | Group 1 | Group 2 | Combined Group |
|---|---|---|---|
| Sample Size | $N_1$ | $N_2$ | $N_1 + N_2$ |
| Mean | $M_1$ | $M_2$ | $\frac{N_1M_1 + N_2M_2}{N_1 + N_2}$ |

| | | | |
|---|---|---|---|
| SD | $SD_1$ | $SD_2$ | $\sqrt{\frac{(N_1-1)\ SD_1^2 + (N_2-1)\ SD_2^2 + \frac{N_1 N_2}{N_1+N_2}\ (M_1^2+M_2^2-2M_1M_2)}{N_1+N_2-1}}$ |

Table S4 Characteristics of each small-molecule metabolite

| Bile acid species | Year | Author | Data soure | Original Unit | If skewed [a] |
|---|---|---|---|---|---|
| Inosine | 2013 | Yun Liu | Digit | mg/L | 0 |
| Adenosine | 2013 | Yun Liu | Digit | mg/L | 0 |

Table S5 The NOS assessment scale for every study

| Study | | Selection | | | | Comparability | Exposure | | | Scores |
|---|---|---|---|---|---|---|---|---|---|---|
| | | S1 | S2 | S3 | S4 | C1 | E1 | E2 | E3 | |
| 2018 | Fanghui Qiu | A* | A* | B | A* | A** | A* | B | B | 6 |
| 2013 | Tingtng Yin | A* | A* | A* | A* | A** | E | A* | C | 7 |
| 2020 | Zheng Zhong | A* | A* | B | A* | A** | A* | A* | C | 7 |
| 2019 | Yitao Li | A* | A* | C | A* | A** | E | B | C | 5 |
| 2020 | Shang lv | A* | A* | B | A* | A** | A* | A* | A* | 8 |
| 2013 | Tie Zhao | A* | A* | B | A* | A** | A* | B | B | 6 |
| 2013 | Meijiao Wang | A* | A* | C | A* | A** | E | B | C | 5 |
| 2013 | Yun Liu | A* | A* | A* | A* | A** | D | A* | C | 7 |
| 2016 | Jiao Chen | A* | A* | B | A* | A** | A* | A* | C | 7 |
| 2021 | Mingmei Zhang | A* | A* | C | A* | A** | E | A* | C | 6 |
| 2016 | Xuefeng Yu | A* | A* | A* | A* | A** | E | A* | C | 7 |
| 2015 | Jiyuan Zhao | A* | A* | C | A* | A** | D | A* | C | 6 |
| 2021 | Tingting Yin | A* | A* | B | A* | B | E | A* | C | 4 |
| 2019 | Fansu Sun | A* | A* | C | A* | A** | C | A* | C | 6 |
| 2014 | Qilin Huang | A* | A* | B | A* | A** | A* | A* | C | 7 |
| 2017 | Tiejuan Shao | A* | A* | B | A* | A** | E | A* | A* | 7 |
| 2021 | Yuqi Chen | A* | A* | C | A* | A** | A* | A* | C | 7 |
| 2017 | Li Cui | A* | A* | B | A* | A** | A* | A* | C | 7 |
| 2020 | Yefei Huang | A* | A* | B | A* | A** | A* | A* | C | 7 |
| 2021 | Lu Kang | A* | A* | B | A* | A** | A* | A* | C | 7 |
| 2011 | Yun Liu | A* | A* | B | B | B | E | A* | C | 3 |
| 2019 | Ying Luo | A* | A* | B | A* | A** | A* | A* | C | 7 |
| 2012 | Yun Liu | A* | A* | B | A* | A** | A* | A* | C | 7 |
| 2022 | Shang Lyu | A* | A* | B | A* | A** | E | A* | C | 6 |
| 2018 | Yannan Zhang | A* | A* | B | A* | A** | A* | A* | C | 7 |
| 2014 | Lisa K. Stamp | A* | A* | A* | A* | A** | A* | A* | C | 8 |
| 2019 | YAN-ZI ZHANG | A* | A* | C | A* | A** | A* | A* | C | 7 |
| 2022 | Shijia Liu | A* | A* | B | A* | A** | A* | A* | C | 7 |
| 2013 | Miao Jiang | A* | A* | A* | A* | A** | A* | A* | C | 8 |
| 2021 | Xia Shen | A* | A* | A* | A* | A** | A* | A* | C | 8 |
| 1999 | Marian Kovalancfk | A* | A* | A* | A* | A** | A* | A* | C | 8 |
| 2018 | Qianqian Li | A* | A* | C | A* | B | E | A* | C | 4 |

Note:

S1: Is the case definition adequate?

S2: Representativeness of the cases.

S3: Selection of Controls.

S4: Definition of Controls.

C1: Comparability of cases and controls on the basis of the design or analysis.

E1: Ascertainment of exposure.

E2: Same method of ascertainment for cases and controls.

E3: Non-Response rate.

* and ** mean score.
